# Seeing the Flaws? Visual Perception of Faces in Individuals Screening Positive for Body Dysmorphic Disorder: An Eye-Tracking Study

**DOI:** 10.3390/jcm15010236

**Published:** 2025-12-28

**Authors:** Łukasz Banaszek, Marta Wojtkiewicz, Monika Rudzińska, Piotr Krysiak, Albert Stachura, Łukasz Mokros, Wiktor Pascal

**Affiliations:** 1 Department of Methodology, Medical University of Warsaw, Banacha 1b Street, 02-097 Warsaw, Poland; 2 Doctoral School, Medical University of Warsaw, 02-091 Warsaw, Poland; 3 Department of Child and Adolescent Psychiatry, Medical University of Łódź, 91-229 Łódź, Poland; 4 Private Practice, Sokolowska 9/31 Street, 01-142 Warsaw, Poland

**Keywords:** body dysmorphic disorder, eye-tracking, auto-perception, facial features

## Abstract

**Background:** Body dysmorphic disorder (BDD) is a psychiatric condition characterized by a preoccupation with perceived appearance flaws. It is highly prevalent among aesthetic surgery candidates and can negatively impact surgical outcomes. The Body Dysmorphic Disorder Questionnaire (BDDQ) is used for BDD screening, but objective validation is limited. This study aimed to determine whether individuals screening positive for BDD exhibit different visual perception patterns of their own and model faces compared to controls, using eye-tracking technology. **Methods:** We conducted a cross-sectional study among 79 participants, including psychiatric patients and medical students. Participants completed the BDDQ and underwent eye-tracking while evaluating standardized photographs of models and their own faces. Gaze fixation patterns were recorded across pre-defined facial areas of interest. Perception and aesthetic assessment differences between the BDDQ-positive and BDDQ-negative groups were studied. **Results:** Participants focused most frequently on the nose, eyes and eyebrows. Compared to model faces, more attention was directed toward their own chin and cheeks. However, BDDQ screening results did not significantly influence fixation patterns or eye-tracking metrics. Psychiatric patients, regardless of BDDQ status, exhibited more numerous and shorter fixations than students. All participants rated model faces as significantly more attractive (i.e., higher aesthetic rating) than their own, with the largest difference observed in the BDDQ-positive group. **Conclusions:** While individuals screening positive for BDD reported lower self-attractiveness, eye-tracking patterns did not differ significantly from those of healthy participants. These findings suggest that BDDQ remains a useful screening tool for subjective dissatisfaction but may not correspond to objective differences in facial visual processing.

## 1. Introduction

Body dysmorphic disorder (BDD) is a mental health disorder wherein patients are excessively preoccupied with perceived flaws in their appearance and spend a significant amount of time daily ruminating on them [1]. To diagnose BDD in line with the Diagnostic and Statistical Manual of Mental Disorders, Fifth Edition (DSM-5), a patient must be concerned with a flaw, which is deemed non-existent or minor by others, engage in compulsive behaviours related to this and be significantly impaired in daily life, with these symptoms not better attributed to an eating disorder [2]. BDD is often comorbid with at least one other condition, such as social phobia, major depression, obsessive compulsive disorder and substance abuse [3,4].

Patients may pursue plastic surgery interventions to change their appearance and alleviate symptoms. BDD affects around 2% of the general population, but in clinical samples of patients undergoing aesthetic surgeries, its prevalence is estimated at 19%. Due to comorbid BDD, the benefit of treatment in this population might be limited [5,6]. Such interventions may even worsen BDD symptoms, and 29–40% of plastic surgeons report legal or physical threats from dissatisfied patients [7,8].

The BDD Questionnaire (BDDQ) is used to screen for BDD. It is self-administered and has a sensitivity of 94–100% and a specificity of 89–90% [9,10]. It focuses on the patient’s concerns about their looks, the time taken thinking about these and how they affect daily life. Although the BDDQ broadly refers to concerns about physical appearance rather than any single body region, facial appearance constitutes one of the most commonly reported and clinically relevant areas of concern in BDD, particularly in populations seeking aesthetic procedures. Given the central role of the face in social interaction and self-identity, facial features are frequently the primary focus of appearance-related distress captured by the BDDQ. A positive result indicates that BDD might be present, and a structured clinical interview with a psychiatrist is needed to confirm the diagnosis. The BDDQ-Aesthetic Surgery (BDDQ-AS) has been devised for patients planning to undergo aesthetic surgery to identify those who would not be satisfied with the outcome [11]. Strengths of the BDDQ include its validation in numerous studies and its self-administration by the patient, allowing for a quick assessment, which takes only 1–5 min. It can therefore be used in a clinical setting, such as during qualification for aesthetic surgery procedures, without the need for psychiatrically trained personnel. It remains the most widely used screening tool by plastic surgeons, and its administration prior to plastic surgery procedures is part of multiple clinical guidelines [12,13,14,15].

Eye-tracking can be used to examine visual perception [16]. It tracks a subject’s gaze, using infrared wavelengths emitted by an eye-tracker placed beside a computer monitor [17]. The number and duration of individual fixations, as well as the eye movement speed and pattern, can be determined. The amount of time spent looking at particular areas may also be recorded. This facilitates an objective analysis, instead of relying on the patient’s own assessment. Eye-tracking has been used widely, from marketing, through aviation, to neuroscience [18]. It has also been used to study mental disorders with abnormal visual processing, e.g., in major depressive disorder, schizophrenia and autism spectrum disorder [19,20]. In BDD, distorted self-perception is thought to be the most pronounced when individuals evaluate their own appearance; therefore, examining visual attention toward one’s own face may provide unique insights into disorder-specific perceptual biases that may not be evident when viewing unfamiliar faces alone. Including both self and other faces allows for differentiation between general abnormalities in facial processing and those specifically related to self-referential perception. It is currently unknown whether a positive BDDQ result is associated with an altered visual perception of faces. Such a link, if present, could aid surgeons in shared decision-making while discussing treatment plans with their patients.

The aim of our study is to determine whether participants who screen positive for BDD have a differing perception of both their own and foreign faces compared with healthy subjects, and consequently, whether the BDDQ is an effective means of identifying patients with abnormal self-perception.

## 2. Materials and Methods

This was a cross-sectional observational study, which was approved by the Institutional Review Board (KB/55/A2023), and informed written consent was obtained from all participants. The report has been prepared in accordance with STROBE guidelines (Supplementary Content S1).

### 2.1. Participants

The study population consisted of a convenience sample recruited from a psychotherapeutic clinic, where psychiatrically stable patients with personality or eating disorders were hospitalized. This cohort was chosen due to a likely high prevalence of comorbid BDD [1]. Moreover, we recruited medical students, who volunteered to take part. The Body Dysmorphic Disorder Questionnaire (BDDQ) screening result and demographic data were collected. The BDDQ was used due to its ease of administration and its prevalence as the most validated screening tool for BDD used by clinicians. The version utilized in this study was prepared and translated by the authors and is available in the Supplementary Content S2. Demographic data included questions about past and present medical history, including any mental disorders, medications, plastic surgeries or aesthetic medicine interventions. Inclusion criteria were an age range from 18 to 65 years, informed consent and completing the BDDQ. Exclusion criteria were vision defects that prevented the participant from using a computer screen without corrective glasses, developmental orofacial defects, a condition after oncological or reconstructive surgery, or a coexisting active mental disorder. Patients who answered “Yes” to question 2 of the BDDQ, intended to detect an isolated eating disorder without a comorbid body dysmorphic disorder, were also excluded from the study.

### 2.2. Experimental Procedure

**Figure 1:**
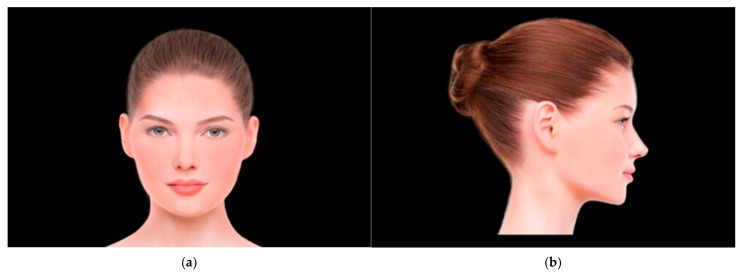
Image of the graphically processed female model face for evaluation: (**a**) The evaluated female model face in frontal view. (**b**) The evaluated female model face in lateral view.

First, we acquired photographs of model male and female faces in a frontal and lateral view using a global stock media provider—iStock. Both models were chosen by the consensus of 3 co-authors of the study. Two model faces were used in total. The selected faces were to have a high level of aesthetics and symmetry, and the use of these faces in the first part of the study was intended to test whether there are any differences in the assessment of the aesthetics of the idealized faces of the models and the participants’ faces. Then, the photographs were graphically processed to eliminate potential bias related to the presence of facial asymmetry or distinguishing marks in the form of skin lesions (Figure 1a,b, Supplementary Content S3). This allowed us to obtain standardized photographs that constituted the material for the study. The experimental protocol was designed using OGAMA Version 5.1 (open gaze and mouse analyzer), an open-source software that enables the creation of an automatically timed slideshow, mapping of the target region and the tracking of visual patterns with an eye-tracker [21].

Each observer was asked to sit on a chair at a standardized distance of 60 cm in front of the computer screen and perform a calibration with an eye-tracking device. The device used was the Gazepoint GP3 HD eye-tracker (Gazepoint, Vancouver, British Columbia (BC), Canada), with a sampling rate of 150 Hz and an accuracy of 0.5–1.0 degrees of the visual angle. The calibration was deemed successful by the device if the observer’s gaze fell within the value of each calibration circle. A chin rest was not used during the experiment. Participants were instructed to maintain a stable head position, and Areas of Interest (AOIs) were defined relative to the facial stimuli displayed on the screen, minimizing the impact of small head movements on gaze allocation. The observers were informed that they would have 20 s to assess the aesthetics of model male and female faces in frontal and lateral view using their eyes. The face stimulus was shown for 13 s. After the allotted time for photograph evaluation, a slide with circles containing scores from 1 to 10 was displayed for 7 s. Scoring required a two-second gaze at one of ten circles, which were located vertically on the right side of the screen to avoid overlapping with the photographs. The time for stimulus presentation was set in the OGAMA software, and slides were displayed automatically.

**Figure 2:**
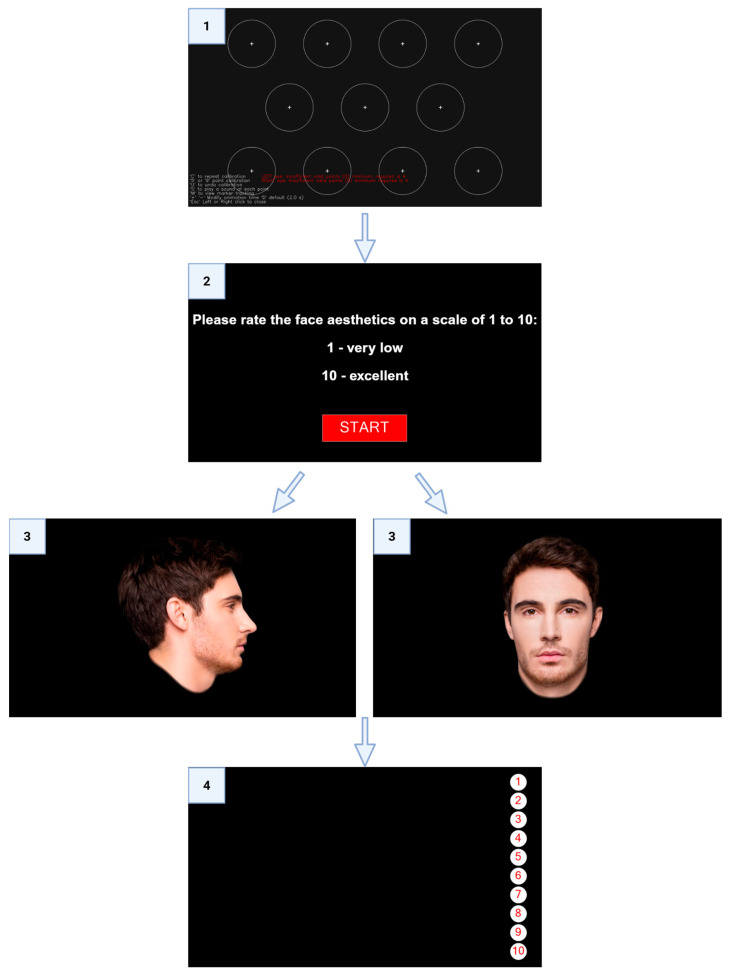
A diagram of the study protocol. 1—11-point calibration process; 2—instructions for the participant (in English for clarity); 3—visual assessment of the faces, 4—aesthetics rating.

After obtaining an adequate quality of the 11-point calibration procedure and ensuring the readiness of the observer, the aesthetics assessment task was displayed. Participants assessed the female face first, then the male model face. The frontal and lateral views of each face were presented sequentially, with scoring taking place after each view. Participants were informed that a score of 1 corresponds to a very low aesthetic appeal, 10 to an excellent appeal, and 5 is a neutral level of attractiveness. In the manuscript, the terms “aesthetics” and “attractiveness” are used interchangeably to refer to the participants‘ rating on the 1–10 scale. The descriptor “excellent” was chosen to guide participants toward a subjective and intuitive rather than a categorical assessment. To avoid bias related to the negative impact of standardized photographs on the assessment of own faces, the evaluation of patients’ photographs took place on the following day, based on the same principles (Figure 2). The order of stimulus presentation was fixed for each participant, with the model-face assessment taking place on day 1 and the own-face assessment on day 2.

**Figure 3:**
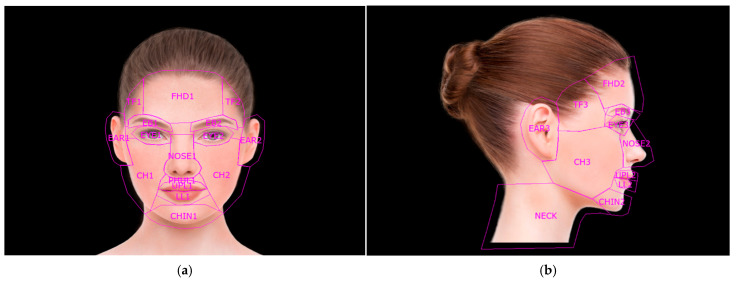
An image of the AOIs chosen for analysis: (**a**) The predetermined AOIs for the frontal view. (**b**) The predetermined AOIs for the lateral view.

An eye-tracking device was used to collect data on gaze fixation patterns using predetermined areas of interest (AOIs) encompassing key facial structures, i.e., forehead, temporal fossa, eyebrows, eyes, ears, nose, cheeks, upper and lower lips, chin, and neck. To maintain coherence between clinical and anatomical points, we designed 27 AOIs in total—16 for the frontal view, 11 for the lateral (Figure 3a,b).

### 2.3. Statistical Analysis

Descriptive statistics included mean ± standard deviations for continuous variables and numbers with percentages for categorical data, unless otherwise indicated. For continuous variables, we first assessed data distribution using QQ plots and used parametric tests (*t*-test) for normally distributed data and nonparametric tests (Wilcoxon, Mann–Whitney U tests) for non-normally distributed data. For categorical data, we used the Chi-square test. The level of agreement between the time spent exploring each AOI was compared for each participant between the self and the same-sex model. We calculated 95% CI (95% CIs) by multiplying the obtained standard errors (SEs) by 3.92. The latter were derived from standard errors of the sample by multiplying by the square root of the sample size. The levels of agreement were visually displayed using Bland–Altman plots [22]. All analyses were performed using R version 4.5.0. Raw data from 3 patients (2 medical students and 1 psychiatric patient; 6% of the total sample) were excluded from analysis due to inadequate visual pathway registration, whereas in 2 patients (2 medical students), a correct calibration could not be performed despite several attempts, and thus, they did not participate in the study.

## 3. Results

The final sample consisted of 79 subjects, including 50 students and 29 psychiatric patients (Table 1). Forty-eight participants (61%) were female, and the mean age of participants was 24.5 years. Most subjects were single, lived in a city with more than 500,000 inhabitants, and had completed secondary school education.

The patient and control groups did not vary significantly in terms of sex, gender, civil status and history of aesthetic surgery. Most differences were observed in categories related to the history of mental disorders or treatment thereof. Patients were treated primarily with SSRIs or SNRIs, less commonly with low-dose antipsychotics. In addition, age and BMI also differed significantly between study groups.

Participants spent a portion of the designated time visually exploring their own (41.5 ± 11.4%) and model (46.2 ± 10.4%) faces, focusing most frequently on the nose (21.8 ± 13.9%), eyes (20.9 ± 13.8%) and eyebrows (19.2 ± 13.8%). Compared to same-sex model faces, males spent more time looking at their own chin (3.9% ± 6.2% vs. 1.9 ± 3.3%, *p* = 0.01) and females at their cheeks (9.6 ± 9.3% vs. 6.6 ± 7.3%, *p* = 0.014) (Table 2).

BDDQ screening result did not influence the perception patterns of either self or model faces, except that individuals who screened positive spent less time exploring their own eyebrows (Table 3).

Moreover, Bland–Altman plots revealed broadly overlapping levels of agreement between perception patterns of self and same-sex model faces for each AOI when both BDDQ groups were compared (Table 4, Supplementary Content S4).

The total number of fixations, their mean duration, and frequency were similar for those who screened positive and negative for BDD. However, participants from the heterogeneous psychiatric cohort had higher total fixation counts (74.9 ± 15.8 vs. 34.3 ± 5.1, *p* < 0.001), more frequent (5.74 ± 1.2 vs. 2.64 ± 0.4) fixations/second, *p* < 0.001) and shorter (118.8 ± 19.7 ms vs. 193.8 ± 43.6 ms, *p* < 0.001) fixations than students (Table 5).

All groups rated the model’s face as significantly more attractive than their own (*p* < 0.001). The largest discrepancy was observed in the BDDQ+ group, MD = −3.3 (−4.68–−1.93), followed by the general psychiatric patients, MD = −2.56 (−3.56–−1.56). Although BDDQ− participants and students also rated their own faces as less attractive than the model’s, the mean differences were smaller, MD = −1.56 and −1.69, respectively (Table 6). Mean differences (MDs) were calculated as self-face ratings minus same-sex model-face ratings; thus, negative values indicate lower attractiveness ratings of one’s own face relative to the model.

## 4. Discussion

In our study, participants, regardless of BDD screening status, predominantly directed their visual attention toward the eyes, eyebrows and nose, dedicating over 60% of their gaze to these regions. This aligns with the notion that socially salient facial features attract more attention [23] and reflects current aesthetic trends (e.g., rhinoplasty and blepharoplasty; ISAPS Global Survey, 2023, https://www.isaps.org/media/rxnfqibn/isaps-global-survey_2023.pdf accessed on 22 November 2025). We also observed increased fixation on the cheeks and chin during self-face viewing, which appeared to be sex-related rather than associated with BDD screening status.

The research on BDD is conflicting, with some studies demonstrating atypical visual processing in affected individuals [24,25], while others report no such differences [26]. Visual patterns in these patients may reflect underlying psychological mechanisms such as perfectionistic schemas or heightened salience of self-related stimuli [25,26,27], whereas imaging studies point to altered dorsal stream processing and a fragmented perception of faces [28].

Contrary to previous studies suggesting altered facial scanning in BDD [24,25,26,28,29,30], our findings did not show significant differences between participants screening positive (BDDQ+) and negative (BDDQ−) for BDD in terms of gaze allocation. Although BDDQ+ participants exhibited a trend toward higher fixation counts and faster scanning patterns, these differences were not statistically significant. This may be due to methodological variations, as our study used the BDDQ, a screening tool with high sensitivity and specificity but relatively low positive predictive value in low-prevalence samples. In non-clinical populations, where BDD prevalence typically ranges between 1.1% and 5.3%, the BDDQ has shown PPVs between 26% and 33% [9,31,32]. Moreover, a heterogeneity of symptoms, subthreshold BDD or misclassification could mask subtle differences.

On the BDDQ, participants in both groups most commonly reported concerns related to the face and its features (e.g., nose, chin, forehead), although concerns were not limited to facial appearance and frequently included other body areas such as the abdomen, breasts and legs. No systematic differences in the distribution of reported body areas were observed between BDDQ-positive and BDDQ-negative participants.

Still, the observed trends resemble patterns described by Toh et al. [24], who reported more effortful and prolonged scanning in BDD patients. Conversely, other studies have described reduced fixations with longer durations, reflecting a focus on negatively perceived attributes [26]. These discrepancies suggest multiple underlying mechanisms—some related to checking and others to avoidance—which may vary across subtypes or levels of severity. Reese et al. [25] and Rossell et al. [27] proposed that such atypical gaze patterns stem from maladaptive beliefs and compulsive checking behaviours.

Moreover, fMRI studies have revealed reduced activation in the dorsal visual stream in BDD patients [28], indicating fragmented face perception, which may underlie the visual strategies described above. However, such results may be confounded by comorbid personality disorders, which are known to co-occur with BDD and could influence attention allocation [33].

In our study, eye-tracking metrics, such as fixation count, scanning speed and saccade ratios, clearly differentiated patients from healthy controls. Rather than reflecting mechanisms specific to body dysmorphic disorder, these patterns may stem from broader psychiatric morbidity, e.g., personality and eating disorders. Previous research has demonstrated that individuals with anorexia nervosa often display atypical saccadic behaviour, which may reflect cognitive inflexibility and altered strategies of face perception [34]. Furthermore, studies including patients with borderline personality disorder (BPD) have reported heightened sensitivity to interpersonal threat cues, with individuals showing rapid orientation toward the eye region of emotional faces [35]. While evidence remains mixed regarding overall fixation durations or counts in BPD, such rapid orienting responses may reflect an underlying hypervigilance—a scanning style also observed across other psychiatric conditions, including post-traumatic stress disorder and generalized anxiety disorder. Across these disorders, hypervigilance has been consistently linked to characteristic alterations in eye-tracking parameters [36].

Taken together, these findings suggest that the gaze observed in our psychiatric cohort may reflect general affective or interpersonal sensitivities, rather than attentional biases uniquely associated with BDD.

Despite similarities in gaze behaviour, all participants rated their own facial attractiveness significantly lower than that of model faces. This effect was most pronounced among BDDQ+ individuals (MD = −3.3, *p* < 0.001) and psychiatric patients (MD = −2.56, *p* < 0.001), consistent with Greenberg et al. [26], who found that BDD patients evaluated their own appearance more negatively and selectively attended to unattractive features. These results support the cognitive-behavioural model of BDD, which posits distorted self-schemas and perfectionism [37,38].

Although Kollei et al. [30] observed that BDD patients attended equally to attractive and unattractive features, our findings suggest a broader negative self-evaluation. Reduced self-attractiveness ratings were observed in participants undergoing psychotherapy regardless of BDD screening status, raising the possibility that personality traits might contribute to appearance-related distortions.

Several limitations should be noted. Our control group consisted of medical students, which limits generalizability. Between-group differences in age, BMI, education and employment may have affected results; for example, higher BMI in the psychiatric group could have influenced self-perception [39]. Accordingly, comparisons between psychiatric patients and medical students should be interpreted cautiously, as these groups were included as reference populations rather than as a demographically matched case–control contrast. Technical factors—such as device calibration, image order and mood variability—may also have influenced outcomes. Additionally, while the BDDQ is a validated instrument [40], its limited specificity may result in false positives, especially in non-clinical samples [41]. While diagnosing BDD using a structured psychiatric interview may have allowed a more accurate identification of subjects with the disorder, our goal was to examine the validity of the BDDQ as a screening tool and its relationship to viewing patterns, to answer the question of whether it is an effective means for plastic surgeons to recognize patients with abnormal visual processing.

It could also be argued that older age and pharmacological treatment in the patients’ population could have confounded the results of our analysis. The age gap, however, was small and unlikely to have had a significant impact on the obtained results. All patients were undergoing psychotherapy at the time of the study, and their mental state was stable after receiving treatment. None of them received benzodiazepines. Therefore, it is unlikely that solely antidepressant treatment or low-dose antipsychotics prescribed at bedtime could have had a clinically meaningful impact on the eye-tracking data.

Regarding stimulus design, the model faces were intentionally graphically standardized to serve as an idealized aesthetic reference, whereas participants’ own facial photographs retained natural variability. Although this difference may introduce variability related to image aesthetics or complexity, such effects would apply equally across BDDQ-positive and BDDQ-negative participants and are therefore unlikely to account for the absence of BDD-specific differences in gaze patterns.

In conclusion, our findings indicate no association between BDDQ screening and visual attention patterns, but a clear link between screening status and self-evaluation of attractiveness. This dissociation highlights the complexity of cognitive distortions in individuals with suspected BDD. Even when gaze behaviour appears similar, the internal evaluation process may differ substantially. These results support the clinical value of combining objective eye-tracking with subjective measures of self-perception when assessing body image disturbances. Taken together, our findings suggest that the BDDQ may primarily capture subjective appearance-related distress rather than BDD-specific neurocognitive or perceptual abnormalities, which may explain its limited association with objective eye-tracking markers.

Future research should explore BDD subtypes, symptom severity and the effects of targeted interventions such as cognitive-behavioural therapy or attention retraining [24,26,30]. It is also important to analyze visual processing in patients with a confirmed diagnosis of BDD, examine the relationship between the disorder and abnormal viewing patterns, and provide insight into the viability of the BDDQ as a screening tool.

## 5. Conclusions

Positive screening for BDD is not associated with major deviations in facial perception patterns. Participants predominantly fixated on the central facial triangle (eyes, nose and eyebrows). They devoted relatively more visual attention to their own cheeks and chin, a pattern that appeared to reflect sex-specific tendencies rather than BDD screening status.

No statistically significant differences in eye-tracking metrics were identified between individuals who screened positive versus negative for BDD. In contrast, psychiatric participants demonstrated markedly different eye-tracking profiles compared to healthy controls, suggesting that eye-tracking metrics may be more sensitive to broader psychiatric morbidity (particularly personality and anxiety disorders) than to BDD-specific characteristics.

These findings highlight the potential utility of integrating objective visual attention measures into the clinical assessment of appearance-related concerns, providing a more nuanced understanding of perceptual and cognitive distortions. BDDQ may help surgeons identify patients vulnerable to negative self-perception, but not mediated by altered visual processing.

## Figures and Tables

**Table 1:** Sociodemographic characteristics of study participants. Continuous variables are presented as means (SD) and categorical variables as numbers (%).

Variable	Patients (*n* = 29)	Control (*n* = 50)	*p*-Value
Sex			0.1517
Male	8 (28%)	23 (46%)	
Female	21 (72%)	27 (54%)	
Gender			0.3298
Male	8 (28%)	22 (44%)	
Female	20 (69%)	27 (54%)	
Non-binary	1 (3%)	1 (2%)	
Age	28.1 (8.5)	22.5 (1.7)	<0.0001
BMI ^1^	23.7 (4.7)	21.6 (2.9)	0.0162
Civil status			0.0715
Single	21 (72%)	29 (58%)	
In a relationship	4 (14%)	21 (42%)	
Married	3 (10%)	0	
Divorced	1 (3%)	0	
Education			0.0004
Primary	2 (7%)	0	
Secondary	18 (62%)	47 (94%)	
Higher (bachelor)	2 (7%)	2 (4%)	
Higher (master’s or further)	7 (24%)	1 (2%)	
Employment			<0.0001
Employed	11 (38%)	3 (6%)	
Unemployed	12 (41%)	0	
Student	2 (7%)	47 (94%)	
(Disability) pension	1 (3%)	0	
No data	3 (10%)	0	
Place of residence			0.0233
Village	4 (14%)	2 (4%)	
Up to 50,000 inhabitants	7 (24%)	3 (6%)	
50,000–100,000 inhabitants	1 (3%)	1 (2%)	
100,000–500,000 inhabitants	0	2 (4%)	
500,000+ inhabitants	17 (59%)	42 (84%)	
Personality disorder			<0.0001
None	5 (17%)	49 (98%)	
Borderline	6 (21%)	0	
Mixed	16 (55%)	0	
Anankastic	1 (3%)	0	
Dependent	1 (3%)	1 (2%)	
Eating disorder ^2^			0.0032
None	22 (76%)	48 (96%)	
Anorexia nervosa	3 (10%)	1 (2%)	
Bulimia	4 (14%)	0	
Mixed	0	1 (2%)	
Comorbidities ^3^			0.0035
None	14 (48%)	41 (82%)	
Mixed anxiety and depressive disorder	8 (28%)	7 (14%)	
Other psychiatric disorders	7 (24%)	2 (4%)	
Psychiatric drugs			<0.0001
Yes	25 (86%)	5 (10%)	
No	4 (14%)	45 (90%)	
Aesthetic surgeries			0.5294
Yes	0	2 (4%)	
No	29 (100%)	48 (96%)	

*p*-values were calculated using Fisher’s exact tests for categorical variables, and *t*-tests for continuous variables. ^1^ Data missing for 3 participants. ^2^ Anorexia nervosa and bulimia include both typical and atypical manifestations. ^3^ Other psychiatric disorders included OCD (*n* = 2), ADHD (*n* = 2), as well as PTSD, alcohol dependence syndrome, other dissociative disorders, other psychotic disorders and somatoform, each with *n* = 1.

**Table 2:** Percentage (%) of time spent on each AOI (calculated as a proportion of the total face stimulus presentation time of 13 s)—differences between self and same-sex model by sexes.

	Male (*n* = 31)			Female (*n* = 48)		
AOI	Self	Model	*p*-Value	Self	Model	*p*-Value
FHD [%]	9.6 ± 10.7	9.1 ± 9.5	0.92	10.3 ± 12.7	13 ± 18.4	0.89
EB [%]	18.5 ± 16.2	19.7 ± 17.4	0.81	15.9 ± 13.2	21.4 ± 15.7	0.06
EYES [%]	17.5 ± 14.4	15.7 ± 14.5	0.79	23.5 ± 15.8	24.1 ± 18.2	0.82
NOSE [%]	21.1 ± 14.3	24.3 ± 16.9	0.72	23.8 ± 16.6	20.9 ± 18.4	0.24
CHIN [%]	4.4 ± 5.9	2.2 ± 3.6	**0.01**	3.7 ± 6.5	1.8 ± 3.3	0.05
EARS [%]	2.8 ± 4	2.5 ± 4.2	0.86	2.2 ± 4.4	2.2 ± 4.7	0.93
CH [%]	11.5 ± 10	9.6 ± 9.6	0.35	8.5 ± 8.6	5.8 ± 7.5	**0.04**
UPL [%]	5.9 ± 11.3	4.6 ± 4.1	0.69	4.4 ± 4.4	5 ± 6.2	0.59
TF [%]	2.4 ± 6	4.1 ± 10.5	0.31	2.2 ± 4.7	1.2 ± 3	0.07
PHUL [%]	3.9 ± 3.4	4.9 ± 5	0.69	3.6 ± 4.3	2.1 ± 2.8	0.11
LL [%]	2.3 ± 2.4	3.2 ± 3.9	0.64	2 ± 2.9	2.3 ± 2.3	0.43

FHD—forehead; EB—eyebrows; CH—cheeks; UPL—upper lip; TF—temporal fossa; PHUL—philtrum; LL—lower lip.

**Table 3:** Percentage (%) time spent on each AOI—differences between screening status by displayed face.

	Self			Same-Sex Model		
AOI	BDDQ−	BDDQ+	*p*-Value	BDDQ−	BDDQ+	*p*-Value
FHD [%]	10.4 ± 11.4	8.7 ± 13.5	0.27	10 ± 10.8	16.4 ± 25.8	0.57
EB [%]	19.1 ± 15.1	9.5 ± 8.5	**0.01**	21.6 ± 17	18.1 ± 13.8	0.55
EYES [%]	20.2 ± 15.9	24.4 ± 13.8	0.21	19.4 ± 16.7	25.8 ± 18.5	0.16
NOSE [%]	21.3 ± 13.5	27.7 ± 21.3	0.33	22.5 ± 17.9	21.5 ± 18.2	0.84
CHIN [%]	3.9 ± 6.6	3.9 ± 4.8	0.52	2.1 ± 3.4	1.6 ± 3.4	0.55
EARS [%]	2.5 ± 4	2.4 ± 5	0.4	2.6 ± 4.9	1.4 ± 2.5	0.79
CH [%]	9.9 ± 9.3	8.6 ± 9.3	0.38	8 ± 9.2	4.9 ± 5.4	0.22
UPL [%]	5.3 ± 8.5	4 ± 4.7	0.59	4.9 ± 5.2	4.7 ± 6.2	0.73
TF [%]	2.2 ± 4.8	2.5 ± 6.4	0.62	2.7 ± 7.8	1.1 ± 3.4	0.38
PHUL [%]	3.4 ± 3.7	4.8 ± 4.8	0.44	3.4 ± 3.9	2.6 ± 4.6	0.37
LL [%]	1.8 ± 2.1	3.3 ± 4.2	0.52	2.8 ± 3.2	1.9 ± 2.4	0.31

**Table 4:** Levels of agreement between exploring own face and the same-sex model’s face measured by the time spent on each AOI. Results presented with 95% confidence intervals. Participants were grouped by the screening status. Positive values indicate spending more time on the same-sex model than the self.

AOI	BDDQ−	BDDQ+
FHD [ms]	−15 (−1169–1139)	665 (−2959–4289)
EB [ms]	300 (−2140–2740)	422 (−946–1790)
EYES [ms]	54 (−2328–2436)	442 (−2022–2906)
NOSE [ms]	42 (−2463–2547)	−31 (−2810–2748)
CHIN [ms]	−90 (−698–518)	−102 (−537–333)
EARS [ms]	−5 (−650–640)	−43 (−694–608)
CH [ms]	−116 (−1412–1180)	−136 (−1212–940)
UPL [ms]	25 (−971–1021)	87 (−544–718)
TF [ms]	38 (−1011–1087)	−33 (−402–336)
PHUL [ms]	−4 (−600–592)	−73 (−900–754)
LL [ms]	71 (−374–516)	−55 (−419–309)

**Table 5:** Eye-tracking parameters by (1) the screening status and by (2) study group.

	**BDD− (*n* = 61)**	**BDD+ (*n* = 18)**	***p*** **-Value**	
Fixation count	45.9 ± 20.5	54.4 ± 24.9	0.27	
Fixation speed	3.6 ± 1.6	4.2 ± 1.9	0.29	
Fixation duration mean	166.9 ± 36.8	172.3 ± 86.7	0.45	
Fixation duration median	127.5 ± 33.4	134.1 ± 55.8	0.78	
Fixation/saccade ratio	516.8 ± 87.5	561.3 ± 82.8	0.07	
	**Patients (*n* = 29)**	**Control (*n* = 50)**	***p*** **-Value**	
Fixation count	74.9 ± 15.8	34.3 ± 5.1	<0.001	
Fixation speed	5.7 ± 1.2	2.6 ± 0.4	<0.001	
Fixation duration mean	118.8 ± 19.7	193.8 ± 43.6	<0.001	
Fixation duration median	87.1 ± 15.9	150.8 ± 28.3	<0.001	
Fixation/saccade ratio	589.4 ± 76.7	494.2 ± 75.3	<0.001	

**Table 6:** Aesthetic evaluation of self vs. model faces across groups.

Group	Scoring (Mean ± SD)		Differences Between Groups (Mean ± 95% CI)	*p*-Value
BDD+	Self (*n* = 19)	4.6 ± 2.37	−3.3 (−4.68–−1.93)	<0.001
	Model (*n* = 20)	7.7 ± 1.23		
BDD−	Self (*n* = 56)	5.5 ± 1.51	−1.56 (−2.08–−1.04)	<0.001
	Model (*n* = 59)	7.1 ± 1.44		
Patients	Self (*n* = 27)	4.7 ± 2.17	−2.56 (−3.56–−1.56)	<0.001
	Model (*n* = 29)	7.2 ± 1.66		
Students	Self (*n* = 48)	5.6 ± 1.5	−1.69 (−2.31–−1.07)	<0.001
	Model (*n* = 50)	7.2 ± 1.25		

## Data Availability

The raw data supporting the conclusions of this article will be made available by the authors on request.

## Supplementary Materials

The following supporting information can be downloaded at: https://www.mdpi.com/article/10.3390/jcm15010236/s1, Supplemental Content S1—STROBE Checklist; Supplemental Content S2—Polish version of BDDQ; Supplemental Content S3—Model faces. Supplemental Content S4—Bland-Altman plots.

## Abbreviations

The following abbreviations are used in this manuscript:

BDD	Body Dysmorphic Disorder
BDDQ	Body Dysmorphic Disorder Questionnaire
AOI	Area of Interest
