# Supplementary material for: Seeing the Flaws? Visual Perception of Faces in Individuals Screening Positive for Body Dysmorphic Disorder: An Eye-Tracking Study
Source: J Clin Med. 2025 Dec 28;15(1):236. doi: 10.3390/jcm15010236 (PMC12786600; doi:10.3390/jcm15010236)
Supplement: Supplementary file 1 [file jcm-15-00236-s001.zip › Supplemental Content 2 - Translated BDDQ Questionnaire.pdf]

Imię i nazwisko ____ Data __________________

Kwestionariusz dotyczy obaw, związanych z wyglądem fizycznym. Proszę przeczytać uważnie pytania i zakreślić odpowiedź, która jest najbardziej prawdziwa. W miejscach na to przeznaczonych, proszę udzielić odpowiedzi pisemnej.

1) Czy martwi się Pan(i) swoim wyglądem? TAK / NIE

- Jeśli tak: Czy myśli Pan(i) o problemach, związanych ze swoim wyglądem często i chciał(a)by Pan(i) myśleć o nich mniej? TAK / NIE

  - Jeśli tak: Proszę wymienić części ciała, których Pan(i) nie lubi:

    ____

    ____

Przykłady nielubianych części ciała to chociażby: skóra (np. pryszcze, blizny, zmarszczki, bladość, zaczerwienienie), włosy, kształt i rozmiar nosa, ust, linii żuchwy, brzucha, bioder itd.; lub niedoskonałości w obrębie rąk, genitaliów, piersi bądź innych części ciała.

<u>UWAGA</u>: Jeśli zaznaczył(a) Pan(i) „Nie" w którymkolwiek z powyższych pytań, tutaj kończy się wypełnianie kwestionariusza. W innym wypadku, proszę kontynuować.

2) Czy Pana/Pani <u>głównym</u> zmartwieniem, dotyczącym wyglądu, jest to, że nie jest Pan(i) wystarczająco szczupły(a) lub że może Pan(i) zbyt mocno przytyć? TAK / NIE

3) W jaki sposób problem, dotyczący Pana/Pani wyglądu wpływa na Pana/Pani życie?

- Czy często to Pana/Panią mocno denerwuje lub smuci? TAK / NIE

- Czy problem ten często powoduje, że rezygnuje Pan(i) z wykonywania czynności lub spotykania się z przyjaciółmi, potencjalnymi partner(k)ami lub sprawia, że ogranicza Pan(i) relacje z innymi ludźmi bądź swoją aktywność społeczną? TAK / NIE

  - Jeśli tak, proszę opisać w jaki sposób: ________________________________

    ____

- Czy powoduje problemy w szkole, pracy lub podczas innych tego typu aktywności? TAK / NIE

  - Jeśli tak, proszę opisać w jakich aktywnościach: __________________________

    ____

- Czy są rzeczy/aktywności, których Pan(i) unika z powodu swojego wyglądu? TAK / NIE

  - Jeśli tak, proszę opisać jakie: ____________________________________

    ____

4) Na co dzień, jak dużo czasu zazwyczaj spędza Pan(i) na myśleniu o swoim wyglądzie? Proszę zsumować cały czas, jaki średnio przeznacza Pan(i) na to w ciągu dnia, a następnie zakreślić najbardziej prawdziwą odpowiedź.

   (a) Mniej niż godzinę dziennie (b) 1-3 godziny dziennie (c) Więcej niż 3 godziny dziennie
