# Supplementary figures and images for: Seeing the Flaws? Visual Perception of Faces in Individuals Screening Positive for Body Dysmorphic Disorder: An Eye-Tracking Study
Source: J Clin Med. 2025 Dec 28;15(1):236. doi: 10.3390/jcm15010236 (PMC12786600; doi:10.3390/jcm15010236)

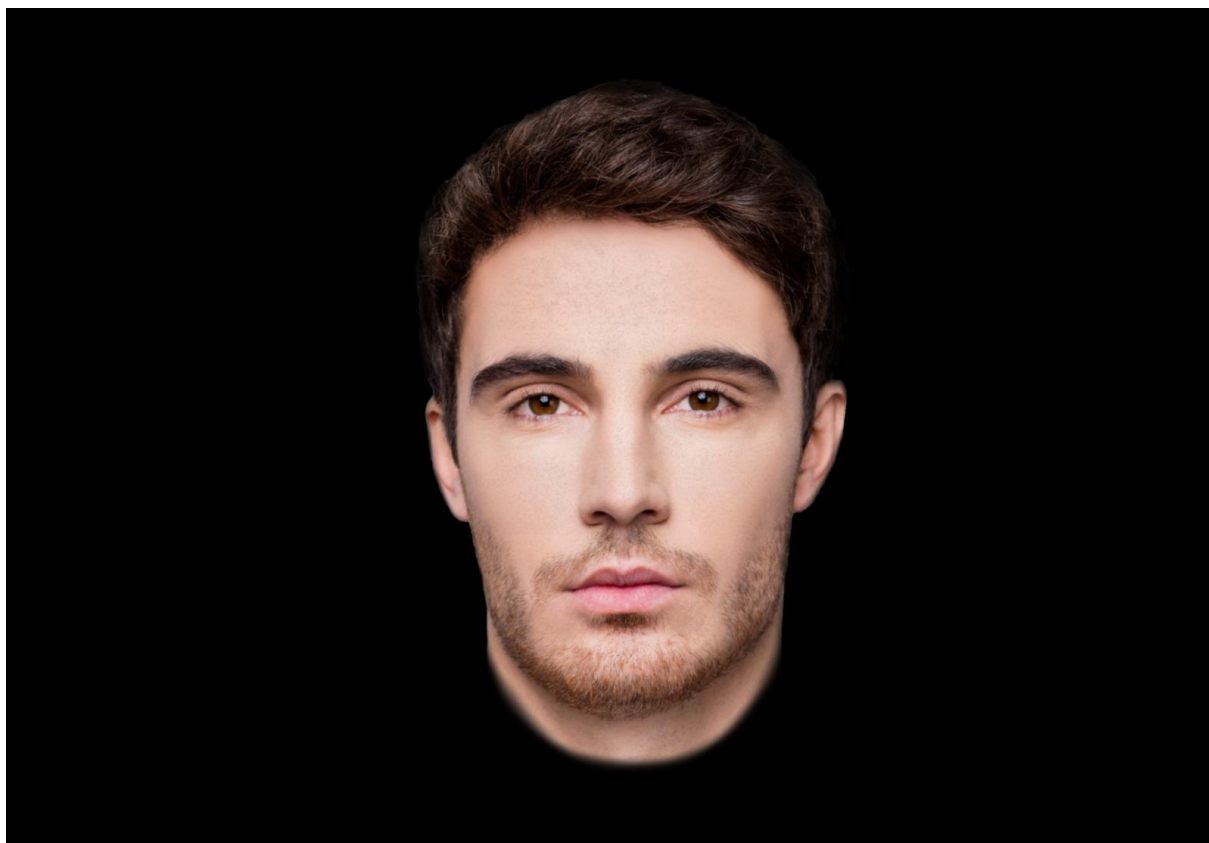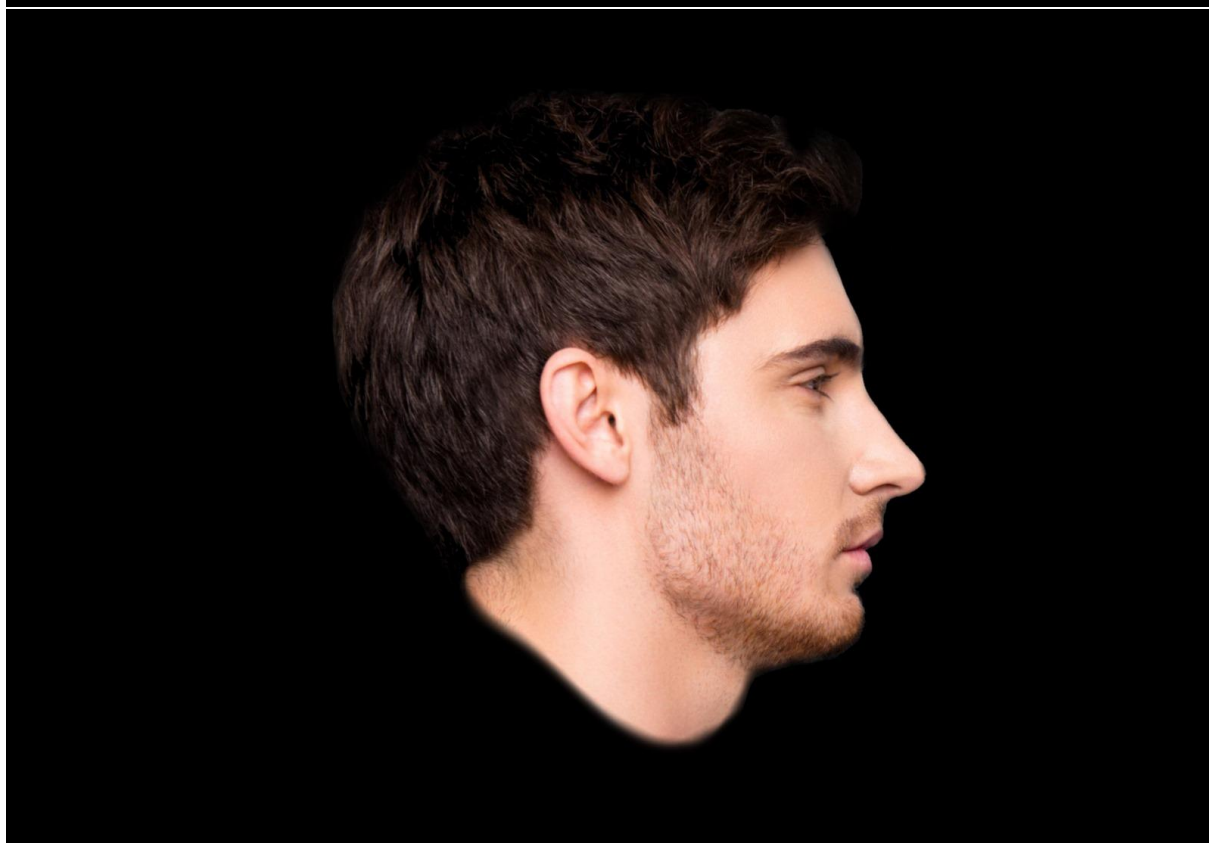

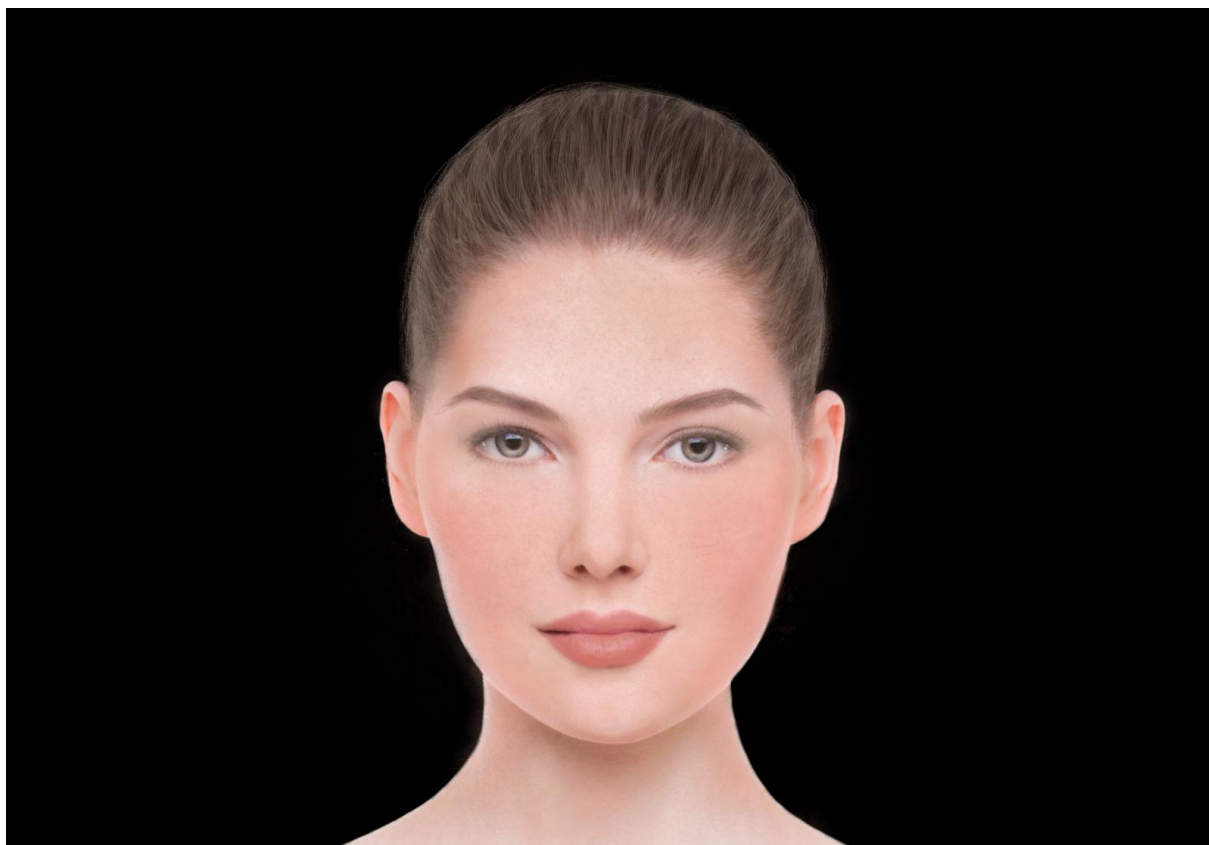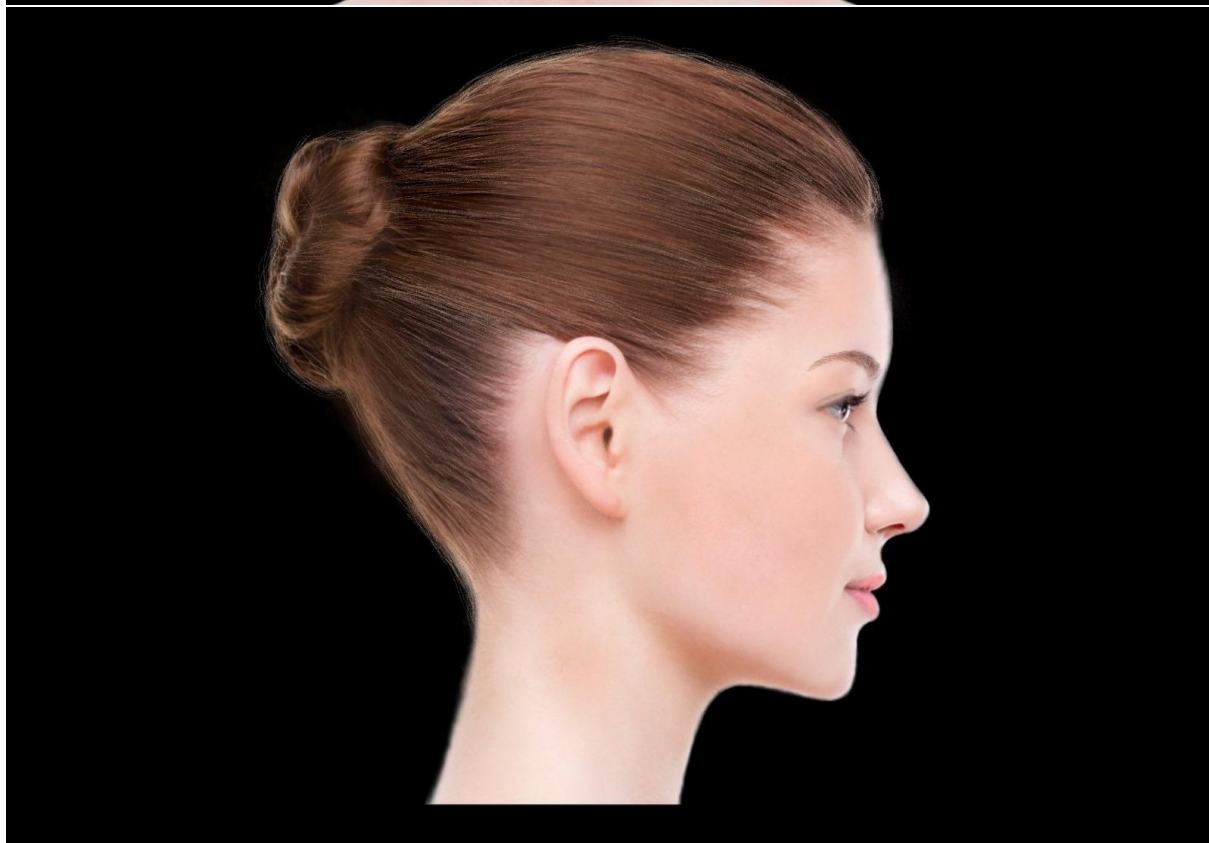

Supplement: Supplementary file 1 [file jcm-15-00236-s001.zip › Supplemental Content 3 - Model faces.pdf]

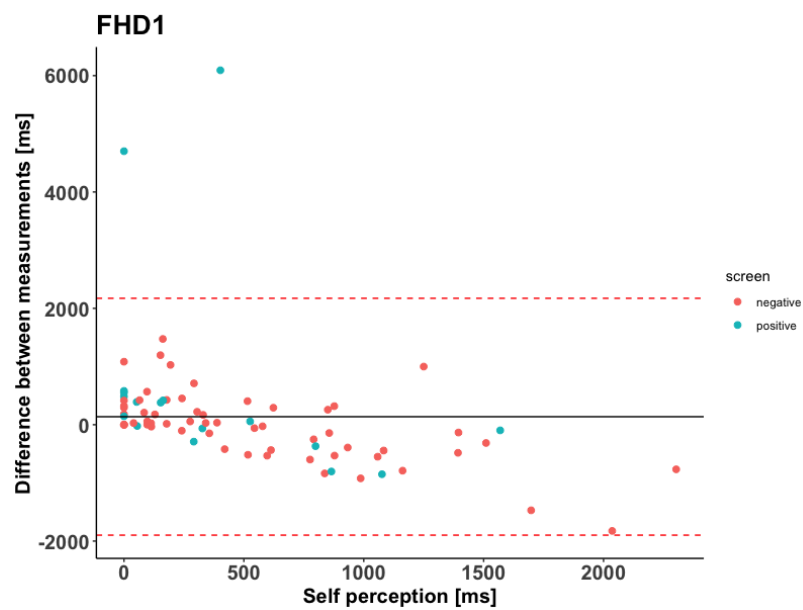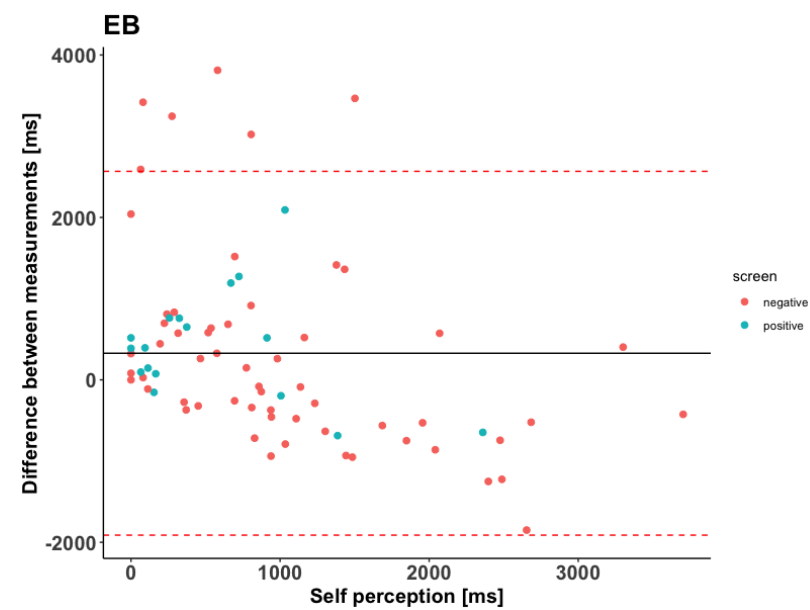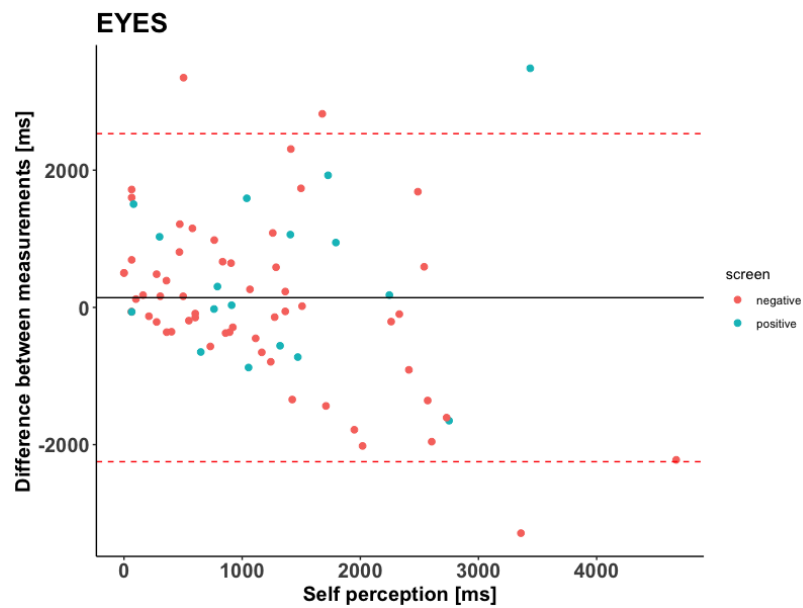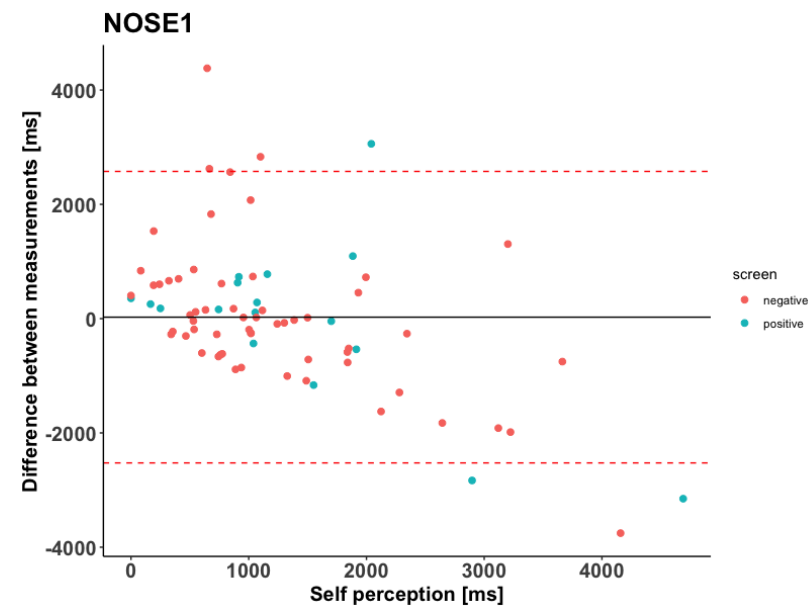

**CHIN1**

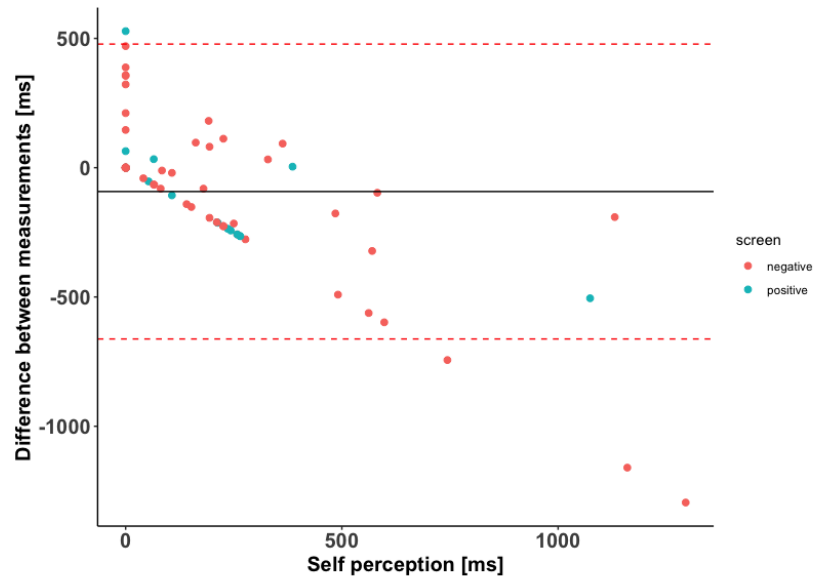

**EARS**

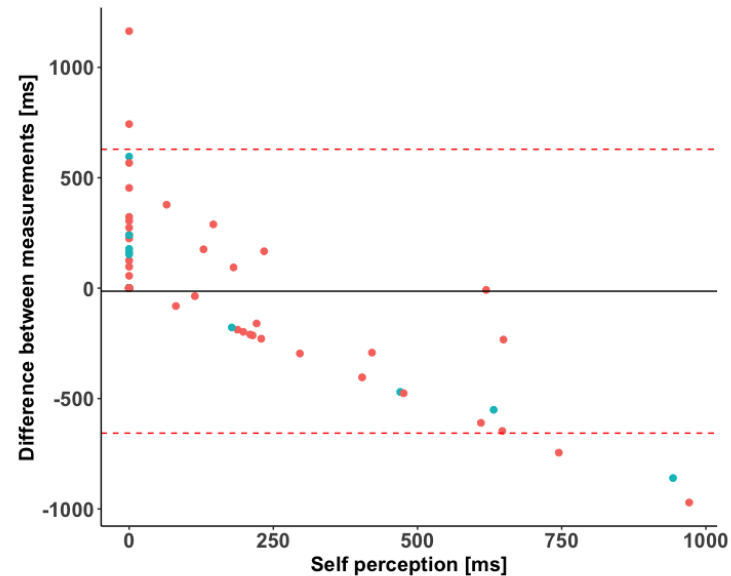

**CH**

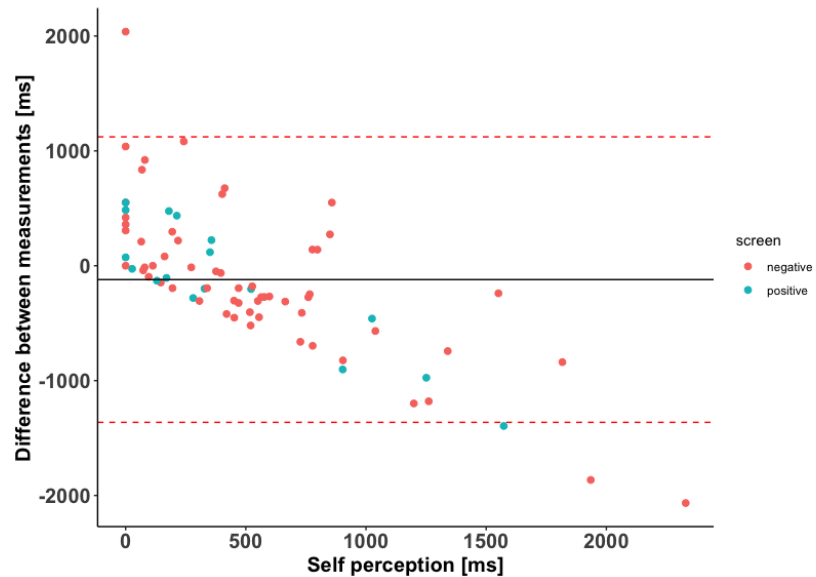

**UPL1**

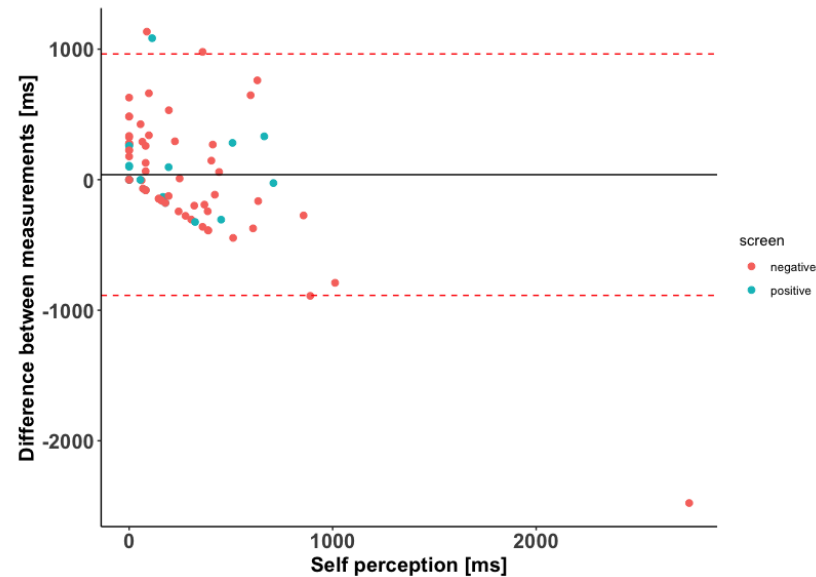

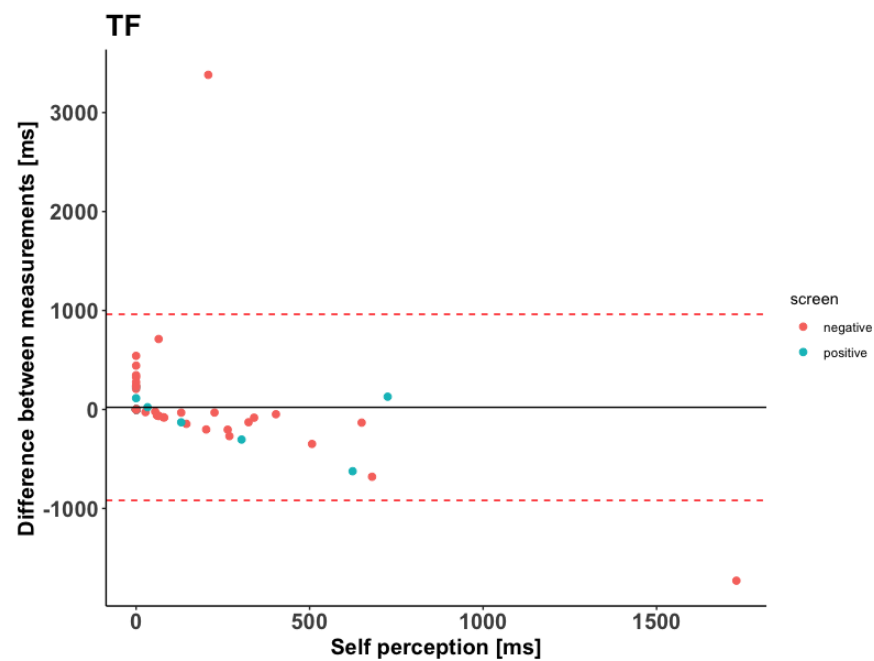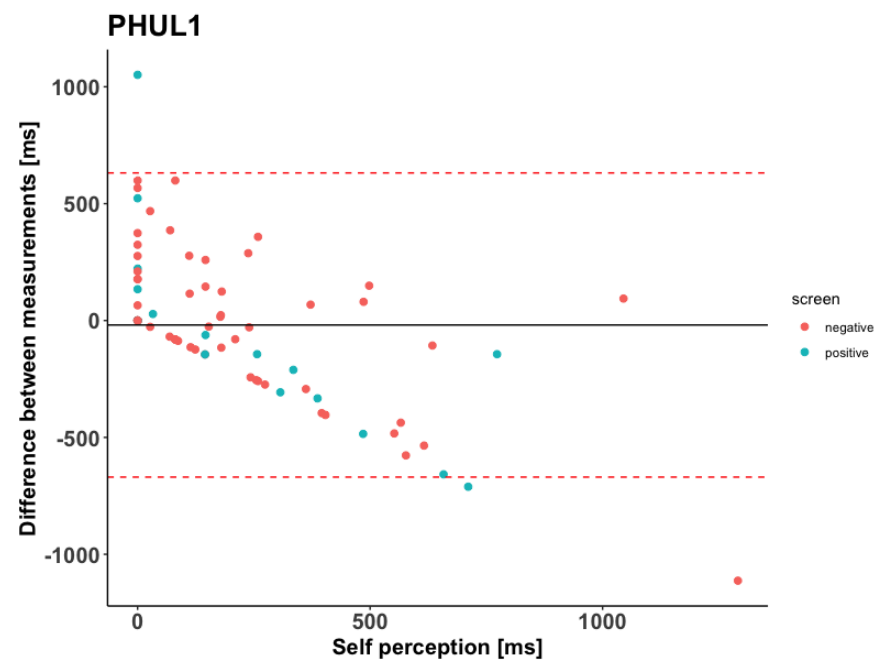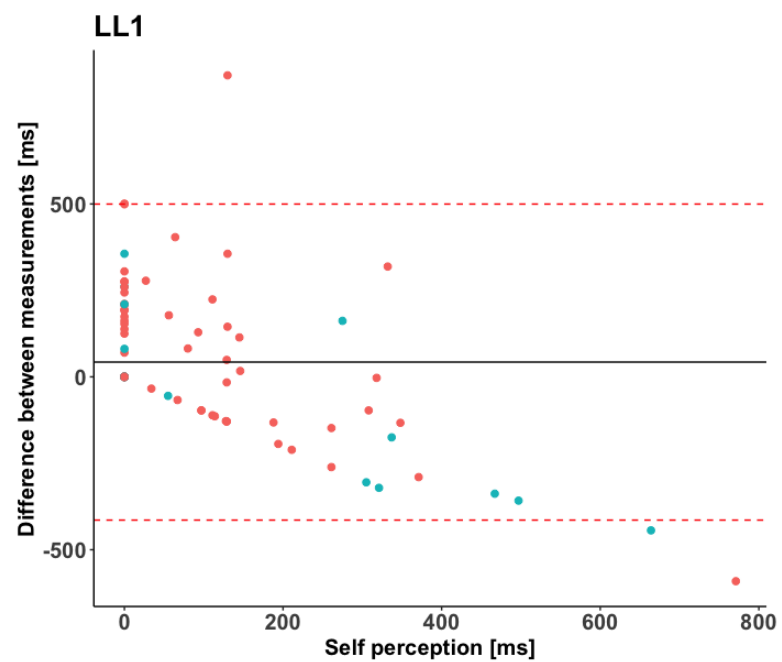

Supplement: Supplementary file 1 [file jcm-15-00236-s001.zip › Supplemental Content 4 - Bland-Altman plots.pdf]
